# Analysis of 8.5 mm Long Dental Implants Provided with Splinted or Solitary Implant Restorations: A 15-Year Prospective Study

**DOI:** 10.3390/jcm13175162

**Published:** 2024-08-30

**Authors:** Jarno Hakkers, Gerdien Telleman, Yvonne C. M. de Waal, Barzi Gareb, Arjan Vissink, Gerry M. Raghoebar, Henny J. A. Meijer

**Affiliations:** 1 Department of Oral and Maxillofacial Surgery, University Medical Center Groningen and University of Groningen, 9700 RB Groningen, The Netherlands; b.gareb@umcg.nl (B.G.); a.vissink@umcg.nl (A.V.); g.m.raghoebar@umcg.nl (G.M.R.); h.j.a.meijer@umcg.nl (H.J.A.M.); 2 Department of Periodontology, Center for Dentistry and Oral Hygiene, University Medical Center Groningen and University of Groningen, 9713 GZ Groningen, The Netherlands; y.c.m.de.waal@umcg.nl; 3 Department of Restorative Dentistry, Center for Dentistry and Oral Hygiene, University Medical Center Groningen and University of Groningen, 9713 GZ Groningen, The Netherlands; g.telleman@umcg.nl

**Keywords:** dental implants, peri-implant health, peri-implantitis, platform switch, splinted restorations

## Abstract

**Background/Objectives:** The long-term effects of implant properties, such as implant length, platform switch, and crown splinting, on peri-implant health require more investigation. Therefore, the aim was to assess the long-term peri-implant health and patient satisfaction in a patient cohort, obtained from two prospective randomized controlled trials, who received 8.5 mm long dental implants, with either splinted or solitary suprastructures and with or without a platform switch, over a period of 15 years. **Methods:** One hundred and twenty-two patients received either one or two 8.5 mm long dental implants (223 dental implants) with and without platform switch, restored with either a solitary (*n* = 89) or a splinted (*n* = 134) restoration in the posterior region. Clinical and radiographical parameters and patient satisfaction were prospectively recorded at 1 month, and 1, 5, and 15 years after the placement of the restoration. Patient satisfaction was recorded with a self-administered questionnaire using a 5-point scale and a visual analog scale (0–10). **Results:** Eighty-one patients with one hundred and fifty-four implants were assessed after a 15-year follow-up. The clinical parameters were low and comparable between the implant types (OsseoTite XP Certain, OsseoTite XP Certain Prevail, NanoTite XP Certain, NanoTite XP Certain Prevail, PalmBeach Gardens, FL, USA) over time. The implants that incorporated a platform switch showed significantly less bone loss than the implants without a platform switch (−0.37 mm, 95% CI −0.69 to −0.05 mm, *p* = 0.024 and β = −0.47, 95% CI −0.80 to −0.14, *p* = 0.006). The implants with splinted restorations experienced more bone loss over time compared to the implants with solitary restorations (0.39 mm, 95% CI 0.15–0.63, *p* = 0.002). Patient satisfaction was high after 15 years. **Conclusions:** All the tested dental implants with 8.5 mm length provide satisfactory 15-year results with regard to the clinical and radiographical parameters as well as patient satisfaction. The platform-matched implants were associated with more bone loss compared to the platform-switched implants, and the implants with splinted crowns portrayed more bone loss than the solitary implant crowns.

## 1. Introduction

The long-term durability of dental implants and their peri-implant mucosal health seem to be dependent on a wide array of factors. Several parameters have been identified in order to prevent the onset of peri-implant disease, such as the cleanability of the prosthesis, bone level, and peri-implant pocket depth [1]. However, studies evaluating the long-term effects of implant characteristics such as implant length, surface topography, platform configuration, and crown design on the health of peri-implant tissues are lacking.

At the time the study was designed, short dental implants, typically defined as implants with lengths < 10 mm, gained increasing attention in implant dentistry due to their potential to address the challenges associated with limited bone height [2]. Despite their shorter length, short dental implants appear to achieve comparable clinical outcomes to longer dental implants over a period of up to 10 years [3,4,5]. However, considerations regarding prosthetic design, crown–implant ratio, and masticatory overload in low-density bone remain paramount for the long-term success of short dental implants since the failures of shorter implants have also been described [6].

The surface topography of dental implants could play a role in determining the biological response of peri-implant tissues [7]. Surface roughness seems to impact collagen fiber orientation after implant placement, suggesting that the surface texture of implants could affect the early stages of soft tissue healing [8]. Comparative studies assessing implants with similar designs but differing surface roughness indicate that there is less average peri-implant bone loss around moderately rough and minimally rough surfaces in comparison to rough surfaces after at least 5 years, but it is improbable that this bone loss can be solely ascribed to surface texture [9]. It was shown that bone-level implants equipped with a platform switch had less peri-implant bone loss than when a platform-matched implant design was used [10]. However, similar performances between platform-switched and platform-matched implants have also been described with regard to clinical and radiographical parameters after 12 months of functional loading [11].

The design of the implant suprastructure could affect peri-implant health when it comes to cleanability. Unfavorable crown contours can contribute to the onset of peri-implant inflammation with peri-implant mucositis and peri-implantitis as a result [12,13]. Another aspect of crown design that has to be considered is connecting neighboring crowns, i.e., splinting. Research indicated that splinting neighboring implant crowns results in a better load distribution along the implant, which could have a beneficial effect on bone stability and implant survival [14,15,16]. However, contradicting results have been presented, reporting that splinting does not prevent, e.g., prosthetic failure [17], and portrays similar survival rates, marginal bone levels, and peri-implant soft tissue conditions as non-splinted implants [18].

In summary, the long-term effects of various implant properties (i.e., implant length, platform switch, and crown splinting) on peri-implant health require further study. Therefore, the aim of this study was to assess the long-term peri-implant health and patient satisfaction in a cohort of patients who received 8.5 mm implants, with either splinted or solitary suprastructures, and with or without a platform switch, 15 years ago.

## 2. Materials and Methods

### 2.1. Study Design, Participants and Setting

This cohort study is a 15-year follow-up analysis based on two prospective randomized controlled trials (122 patients and 223 implants) on the impact of platform switching and implant surface topography, prospectively assessing the peri-implant health of 8.5 mm long dental implants [19,20]. All the implants were placed in the premolar and molar regions of the maxilla and mandible. Four case specifications were identified:

Patients receiving one or two neighboring 8.5 mm long dental implants with a non-platform-switched design restoration (OsseoTite XP Certain, Biomet 3i, PalmBeach Gardens, FL, USA);
Patients receiving one or two neighboring 8.5 mm long dental implants with a platform-switched design restoration (OsseoTite Certain Prevail, Biomet 3i, PalmBeach Gardens, FL, USA);
Patients receiving one or two neighboring 8.5 mm long dental implants with a non-platform-switched design restoration (NanoTite XP Certain, Biomet 3i, PalmBeach Gardens, FL, USA);
Patients receiving one or two neighboring 8.5 mm long dental implants with a platform-switched design restoration (NanoTite Certain Prevail, Biomet 3i, PalmBeach Gardens, FL, USA).

Data on the implant and prosthesis survival, and patient satisfaction were prospectively collected. Patient inclusion, treatment, and follow-up appointments took place in the Department of Oral and Maxillofacial Surgery at the University Medical Center Groningen (UMCG) in the Netherlands. The Medical Ethics Review Board of the UMCG approved the research protocol of the 15-year extension follow-up study (METc nr. 2022/434). The study was registered in ClinicalTrials.gov (NCT05650099) and conducted in accordance with the Declaration of Helsinki. Written informed consent was obtained from all the participants before enrolment. This study was written based on the STROBE guidelines.

### 2.2. Outcomes and Parameters

The surgical and restorative procedures are described in the initially performed studies [19,20]. Data were recorded at baseline (T_pre_), after the placement of the implant crown (T_5_), 1 year postoperatively (T_12_), 5 years postoperatively (T_60_), and 15 years postoperatively (T_180_). Periodontal probing depth (PD) was measured in millimeters (mm) using a Hu-Friedy^®^ PCPUNC156 periodontal probe. Any subsequent bleeding on probing was recorded using the Sulcus Bleeding Index [21] and the signs of peri-implant inflammation were recorded using the Gingival Index [22]. Plaque was scored in accordance with the Plaque Index [21]. The bone levels were assessed using peri-apical radiographs (Planmeca Intra X-ray unit; Planmeca, Helsinki, Finland) and were taken using a paralleling technique. The measurement of peri-implant bone loss was carried out using the DICOM software (DicomWorks 1.5). The calibration of each radiograph was performed using a 3-point reference scale based on the known implant length and/or diameter. Subsequently, the bone level variances were determined for both the mesial and distal regions of the implant. Patient satisfaction was evaluated through a self-administered questionnaire to be completed at T_pre_, T_5_, T_60_, and T_180_. The questionnaire consisted of inquiries or statements to be rated on a five-point scale, ranging from “very dissatisfied” and “not in agreement” (score 1) to “very satisfied” and “in agreement” (score 5). The questionnaire covered aspects such as aesthetics, function, and treatment procedures. Additionally, the patients were asked to assess their overall oral satisfaction, specifically regarding areas where teeth were replaced by implants, using a 10-point rating scale from 0 to 10, where 10 represented the highest satisfaction level. Complications regarding the implant restoration were recorded based on visual examination, recordings based on previous visits, and live patient anamnesis.

### 2.3. Complications

Complications were subdivided into biological complications and restorative complications. Biological complications, i.e., peri-implant mucositis and peri-implantitis, were defined in accordance with the diagnostic criteria stipulated by the 2017 World Workshop [23] on the classification of periodontal and peri-implant diseases and conditions, taking the baseline measurements into account:

Diagnosis of peri-implant mucositis:

- The presence of bleeding and/or suppuration on gentle probing with or without increased probing depth compared to previous examinations.
- The absence of bone loss beyond the crestal bone level changes resulting from initial bone remodeling.

Diagnosis of peri-implantitis:

- The presence of bleeding and/or suppuration on gentle probing.
- Increased probing depth compared to previous examinations.
- The presence of bone loss beyond the crestal bone level changes resulting from initial bone remodeling.

Restorative complications were scored based on clinical examination, previous recordings, and patient anamnesis, and could consist of, e.g., chipping/fracture of the restorative material or screw loosening.

### 2.4. Statistical Analysis

The nominal and ordinal outcome measures were presented as frequencies and corresponding percentages. The distribution of the continuous variables was checked visually using histograms and QQ plots as well as using the Shapiro–Wilk test. Normally distributed continuous data were described as mean and standard deviation (SD). Skewed distributed continuous variables were presented as median with interquartile range (P25-P75). The Wilcoxon signed-rank test was performed for the within-person comparison (i.e., related samples) of the skewed distributed variables (e.g., improvement in satisfaction score compared to the pre-treatment score). The within-person comparison (i.e., related samples) of the dichotomous outcomes was performed using the McNemar mid-p test. Since patients with more than one implant were included (i.e., related samples) with multiple measurements over time, multivariable linear mixed effect models (LMM) and multivariable mixed effects logistic regression models were fitted using restricted maximum likelihood estimations. The multivariable models included the fixed effects of the baseline value, follow-up in months, implant type, implant diameter, and restoration (i.e., splinted versus solitary restoration). In these models, the effect of these variables on marginal bone loss, plaque index, gingival index, bleeding index, probing depth, peri-implant mucositis, and peri-implantitis was assessed while accounting for within-person variability and repeated measurements. The multivariable models were fitted using a stepwise backward elimination approach. A variable was considered a confounder whenever the regression coefficient of implant type, implant diameter, and/or restoration (i.e., splinted versus solitary restoration) by 10% or more or whenever a variable was a known confounder based on the prior literature. The included random effects were the patient and implant location (i.e., random intercepts) with an unstructured covariance matrix. The interaction between the baseline and follow-up value (baseline value*follow-up) was tested for model improvement but did not significantly improve the estimated models and was, thus, excluded from the models. Model improvement was tested using likelihood-ratio tests. The following assumptions underlying the final mixed effect models were tested and verified: (1) posterior predictive check (tested by simulating the replicated data under the fitted model and then comparing these to the observed data), (2) the predictor variables are linearly associated with the outcome (tested by plotting the residuals versus the fitted values), (3) the homogeneity of the variance (tested by plotting the square root of the standardized residuals versus the fitted values), (4) influential observations (tested by plotting the standardized residuals versus the leverage and assessing whether the observed values fall within the Cook’s distance), (5) normally distributed residuals (tested using QQ-plots of the residuals), and (6) the independence of the data points of different participants (by design: the study participants are independent, i.e., unrelated) [24]. In addition, the log transformation of the outcome data was performed to assess whether the model improved. However, this was not the case for any of the fitted models. All the models yielded estimated regression coefficients (β) and 95% confidence intervals (CI). The effect estimated by the mixed effect logistic regression models was converted to the corresponding odds ratio (OR) with 95% CI. Statistical comparisons were performed using the type III Analysis of Variance (ANOVA) test with Satterthwaite’s method to estimate the degrees of freedom. Missing data (e.g., due to drop outs) were not imputed since mixed models can handle these data well (i.e., including the available data even when cases have some missing data), and there is no gain from handling missing data (e.g., by multiple imputations) before performing mixed-model analysis on longitudinal data [25]. The statistical analyses were performed in R, version 4.0.5 (R Core team) using the lme4- and lmertest-packages. A significance level of *p* < 0.05 was chosen for all the analyses. Bonferroni adjusted was applied in case of multiple comparisons.

## 3. Results

**Figure 1:**
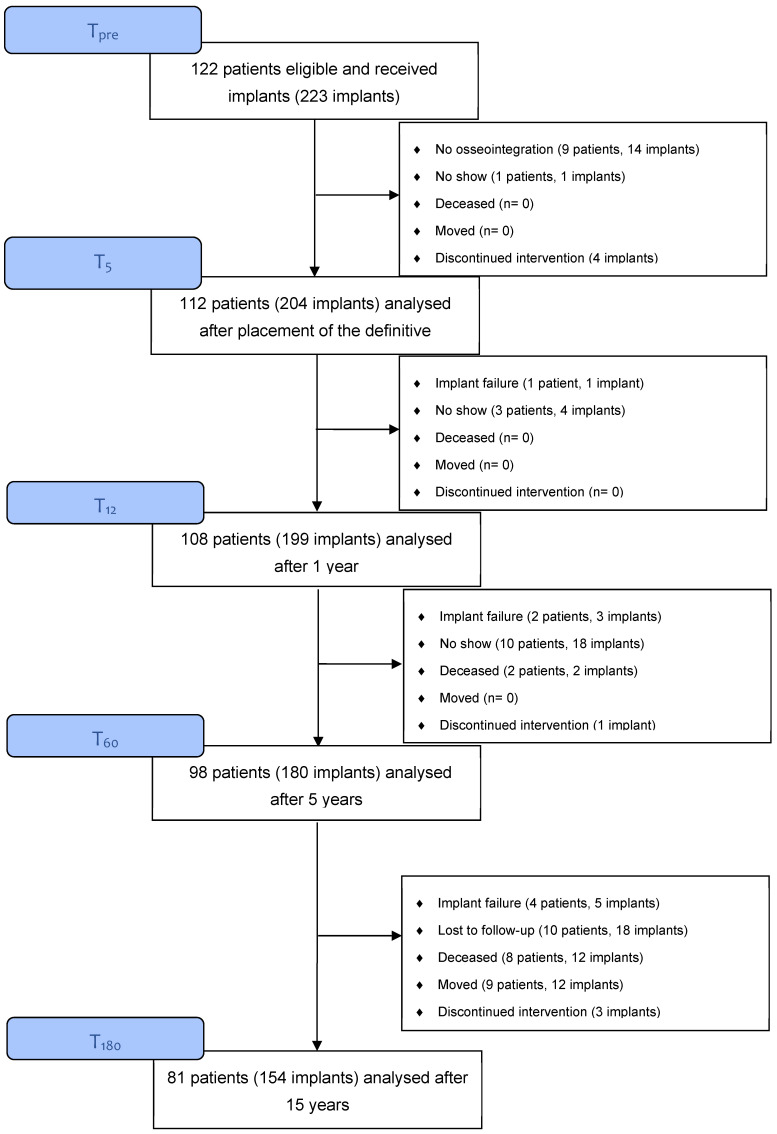
Flow chart.

Table 1 shows the patient characteristics. These did not significantly differ between the groups and were, therefore, presented collectively. At T_pre_, 122 patients with 223 implants were included. A total of 112 patients with 204 implants received a restoration. At T_180_, 81 patients with 154 implants could be analyzed. The others had lost an implant, were deceased, or lost to follow-up (Figure 1). Table 2 shows the clinical parameters over time. Table 3 shows the radiographical bone change on the timepoints T_12_, T_60_, and T_180_.

When evaluating the peri-implant health, 9 out of the 204 implants that received a definitive restoration (4.4%) were lost due to peri-implantitis. At T_180_, 25 (16.2%) of the remaining 154 implants were diagnosed with peri-implant mucositis, and 8 (5.2%) implants with peri-implantitis. A total of 28 (13.7%) of the 204 implants experienced restorative complications, being screw loosening (2 implants) and porcelain chipping (26 implants) during the follow-up period. Table 4 shows the results regarding the association between implant type, implant diameter, splinted restorations, and the presence of peri-implant mucositis and peri-implantitis on T_180_. These implant and restoration characteristics were not significantly associated with peri-implant mucositis or peri-implantitis (Table 4).

Table 5 shows the effect of implant type, implant diameter, and splinted restorations (yes/no) on bone loss, plaque index, bleeding index, gingiva index, and peri-implant pocket depth. In the multivariable model, the implant types OsseoTite Certain Prevail (β = −0.37, 95% CI −0.69 to −0.05, *p* = 0.024) and NanoTite Certain Prevail (β = −0.47, 95% CI −0.80 to −0.14, *p* = 0.006) both showed significantly less bone loss compared to OsseoTite XP Certain (reference). Furthermore, splinted restorations were associated with more bone loss compared to no splinted restoration (β = 0.39, 95% CI 0.15–0.63, *p* = 0.002). Of all the clinical outcomes, the NanoTite XP Certain implant was associated with a lower bleeding index compared to the other implant types (β = −0.16, 95% CI −0.33 to 0.00, *p* = 0.049).

Table 6 shows the patient satisfaction at different follow-up assessments. The patient satisfaction significantly increased for all the questions during the follow-up measurements compared to the pre-operative scores (*p* < 0.001). The patient satisfaction remained high and stable at each follow-up assessment.

## 4. Discussion

This study analyzed long-term peri-implant health and patient satisfaction in a cohort of patients who received 8.5 mm implants, with and without platform switching, and either splinted or solitary suprastructures, over a period of 15 years. In short, this cohort of patients shows satisfactory clinical, radiographical, and patient-experienced long-term results.

When it comes to implant length, dental implants < 10 mm seem to exhibit similar results when compared to longer dental implants, but might fail at an earlier stage if a failure does occur [26]. Moreover, it seems that implant lengths shorter than 8 mm might present a greater risk of failure [6]. In this cohort, 14 out of the initial 223 implants (6.3%) experienced a failure of osseointegration. Furthermore, 9 (4.4%) out of the 204 implants that received a restoration were lost due to peri-implantitis, and 28 (13.7%) of the 204 implants experienced restorative complications. Comparable findings were observed in the 5–10 year follow-up results by Lai et al. (2013) where 18 (7.8%) of the short dental implants faced biological complications and 29 (12.6%) of the implants had undergone technical complications [27]. Also, when it comes to patient satisfaction, the results are comparable with the initial clinical trials and follow-up in which patient satisfaction did not seem to alter over time [19,20].

In this study, the implant type (encompassing both the implant surface and the platform switch) was associated with bone loss, where bone loss seems to be higher around the implants that had a platform-matched design. The advantages of platform-switched implant designs have been extensively studied, yielding varying outcomes in the available literature. Taheri et al. (2020) concluded that bone-level, platform-switched implants demonstrated better marginal bone stability compared to tissue-level implants or bone-level, platform-matched implants [28]. Santiago et al. (2016) performed a systematic review and meta-analysis in which they concluded that a reduction in bone loss with platform-switch implants was observed in the included randomized controlled trials, implants in the maxilla, and implants in the mandible when compared to regular platform implants [10]. Arai et al. (2024) also recommend the use of a platform-switched implant design to prevent marginal bone loss [29]. However, Uraz et al. (2020) reported similar performances between platform-switched and platform-matched with regard to clinical and radiographical parameters implants after 12 months of functional loading [11]. Our findings support the favorable bone stability of a platform-switched implant design, observed in this study after the long-term follow-up (i.e., 15 years).

The prevalence of peri-implantitis is highly dependent on the criteria used. Several systematic reviews and meta-analyses report data on the prevalence of peri-implantitis, with numbers varying between 19.5% and 42% [30,31]. The factors that can influence the course and progression of peri-implantitis, such as implants placed in native bone or augmented bone, are often not taken into account. In the systematic review and meta-analysis performed by Salvi et al. (2018), it was concluded that the prevalence of peri-implantitis at the patient level was estimated to have a weighted mean of 10.3% (95% CI: 4–17%) for pristine sites and 17.8% (95% CI: 0–37%) for augmented sites [32]. Based on the diagnostic criteria from the 2017 World Workshop, 25 (16.2%) of the remaining 154 implants were diagnosed with peri-implant mucositis, and 8 (5.2%) implants with peri-implantitis. These results are somewhat lower than expected. If the implants that were removed based on the diagnosis of peri-implantitis during the entire follow-up are also taken into account, this number increases to 17 (8.3%) out of the 204 implants that received a definitive restoration, which comes closer to the figure presented by Salvi et al. (2018) [32]. It should be noted that the diagnostic criteria based on which an implant was removed were different since several implants in this study were removed prior to 2017. However, there are no definitive guidelines specifying at what stage or severity of peri-implantitis an implant should be removed.

Neighboring implants received crowns that were connected to each other. The rationale for this was that there were indications that splinting the implant crowns would result in a better load distribution along the implant, which could have a beneficial effect on bone stability and implant survival [14,15,16]. However, more recent literature seems to highlight different results, stating that splinting does not seem to prevent, e.g., prosthetic failure [17], and portrays similar survival rates, marginal bone levels, and peri-implant soft tissue conditions when compared to non-splinted implants [18]. Even in a vulnerable state, e.g., directly after implant placement with immediate provisionalization, splinting might be unnecessary when canine guidance is present, along with a stable occlusion [33]. In our study, implant/prosthesis survival was not correlated with prosthesis design, and marginal bone loss significantly decreased over time around implants with splinted crowns. The available literature on the long-term effects of splinted constructions on short implants is limited. Esposito et al. (2023) conducted a study comparing 6 mm implants under the same fixed implant prosthesis with two individual implants supporting single crowns in the mandible [34]. It was concluded that the prognosis of short dental implants in the mandible may not be influenced by splinting them via the fixed prosthesis. It must be noted that this study only examined implants in the mandible over a 5-year period of time.

There are several limitations with regard to this study. For one, the comparison between splinted and solitary implants could be distorted due to the fact that solitary implants did not have a neighboring implant with a solitary implant crown. Two neighboring implants with two non-splinted crowns would have enhanced the clinical and radiographical analysis with regard to the clinical effects of crown connection on the peri-implant tissues. Besides, it is also important to recognize that the comparison of bone loss occurred in a situation where two implants were placed adjacent to each other, versus a single implant with a neighboring tooth. The latter could imply that the observed bone loss is not necessarily causally related to the fact that the crowns are splinted, but rather due to the presence of two adjacent implants. Given that, at the time of implantation, the guidelines for inter-implant distance were followed (>3 mm distance between two implants), the effect of this should be limited. To the best of our knowledge, this is the first study prospectively assessing implant characteristics in relation to the health of peri-implant tissues with a follow-up of 15 years.

## 5. Conclusions

Within the limitations of this study, 8.5 mm implants in the posterior region are accompanied by satisfactory long-term results with regard to clinical and radiographical parameters, and patient satisfaction. The platform-matched implants were significantly associated with a higher level of bone loss when compared to the platform-switched implants during the follow-up period of 15 years, and the implants with splinted crowns portrayed more bone loss than the solitary implant crowns.

## Figures and Tables

**Table 1:** Patient characteristics.

Characteristics	
Number of patients	122
Mean age at implant placement (SD, years)	49.1 (12.0)
Female/male	94/28
Number of implants	223
Implant position maxilla (P1/P2/M1/M2)	12/33/52/8
Implant position mandible (P1/P2/M1/M2)	3/41/61/13
Number of solitary implants	89
Number of splinted implants	134
Implant diameter 4.1 mm (*n* implants)	158
Implant diameter 5.0 mm (*n* implants)	65
Surface characteristics (OsseoTite/NanoTite)	89/134
Number of restorations with platform switching (yes/no)	114/109

**Table 2:** Plaque, bleeding, and gingival index score (%) and mean deepest pocket depth (mm, SD).

	T_5_	T_12_	T_60_	T_180_
Plaque index score 0; no detection of plaque	89.2	73.7	71.2	98.0
Plaque index score 1; plaque on probe	10.8	22.2	23.7	0.7
Plaque index score 2; plaque seen by naked eye	0	4.1	5.1	1.3
Plaque score 3; abundance of soft matter	0	0	0	0
Bleeding index score 0; no bleeding	60.0	60.1	50.3	78.4
Bleeding index score 1; isolated bleeding spots	38.9	37.4	41.8	14.2
Bleeding index score 2; confluent line of blood	1.1	2.5	7.9	7.4
Bleeding index score 3; heavy or profuse bleeding	0	0	0	0.0
Gingival index score 0; normal mucosa	91.6	94.4	86.4	94.6
Gingival index score 1; mild inflammation	8.4	5.6	13.6	5.4
Gingival index score 2; moderate inflammation	0.0	0.0	0.0	0.0
Gingival index score 3; severe inflammation	0.0	0.0	0.0	0.0
Mean deepest pocket depth	3.4 (1.2)	3.2 (1.1)	3.6 (1.3)	2.8 (1.0)

**Table 3:** Changes in peri-implant bone loss over time.

Bone Change (mm)	T_12_	T_60_	T_180_
Mean (SD)	1.2 (0.8)	1.1 (0.9)	1.4 (1.2)
>−2.0 mm (*n* implants (% implants))	37 (18.8)	34 (19.2)	40 (26.7)
−1.5 to −2.0 mm (*n* implants (% implants))	36 (18.3)	26 (14.7)	16 (10.7)
−1.0 to −1.5 mm (*n* implants (% implants))	40 (20.3)	23 (13.0)	26 (17.3)
−0.5 to −1.0 mm (*n* implants (% implants))	38 (19.2)	38 (21.5)	34 (22.7)
0.0 to −0.5 mm (*n* implants (% implants))	46 (23.4)	56 (31.6)	34 (22.7)

**Table 4:** Outcomes regarding implant characteristics in relation to peri-implant mucositis and peri-implantitis.

Predictors	Peri-Implant Mucositis		Peri-Implantitis	
	Odds Ratios (95% CI)	*p*	Odds Ratios (95% CI)	*p*
Implant type (ref: OsseoTite XP Certain)				
OsseoTite Certain Prevail	2.14 (0.00–1794.65)	0.825	0.62 (0.00–73,429.32)	0.935
NanoTite XP Certain	1.45 (0.00–1687.10)	0.918	0.00 (0.000–Inf)	0.990
NanoTite Certain Prevail	0.59 (0.00–1660.80)	0.896	0.63 (0.00–102,412.96)	0.940
Implant diameter (ref: 4.1 mm)				
5.0 mm	0.74 (0.00–232.75)	0.920	5.66 (0.00–261,784.25)	0.752
Splinted (ref: no)				
Yes	0.67 (0.00–139.71)	0.884	3.60 (0.00–517,583.95)	0.833

**Table 5:** Outcomes regarding implant characteristics in relation to clinical and radiographical parameters.

Predictors	Bone Loss		Plaque Index		Bleeding Index		Gingiva Index		Peri-Implant Pocket Depth	
	β (95% CI)	*p*	β (95% CI)	*p*	β (95% CI)	*p*	β (95% CI)	*p*	β (95% CI)	*p*
Implant type (ref: OsseoTite XP Certain)										
OsseoTite Certain Prevail	−0.37 (−0.69 to −0.05)	0.024	0.12 (−0.00 to 0.25)	0.056	0.00 (−0.16 to 0.16)	0.999	0.05 (−0.03 to 0.12)	0.221	0.12 (−0.22 to 0.47)	0.484
NanoTite XP Certain	−0.10 (−0.44 to 0.25)	0.580	0.06 (−0.08 to 0.19)	0.405	0.16 (−0.33 to 0.00)	0.049	0.01 (−0.07 to 0.09)	0.855	0.02 (−0.36 to 0.40)	0.916
NanoTite Certain Prevail	−0.47 (−0.80 to −0.14)	0.006	0.11 (−0.02 to 0.24)	0.083	0.13 (−0.28 to 0.03)	0.114	0.01 (−0.07 to 0.08)	0.886	−0.09 (−0.46 to 0.28)	0.628
Implant diameter (ref: 4.1 mm)										
5.0 mm	−0.19 (−0.43 to 0.06)	0.130	−0.03 (−0.13 to 0.06)	0.510	0.00 (−0.12 to 0.12)	0.978	−0.02 (−0.08 to 0.04)	0.451	0.26 (0.00 to 0.53)	0.051
Splinted (ref: no)										
Yes	0.39 (0.15 to 0.63)	0.002	0.06 (−0.04 to 0.15)	0.248	0.08 (0.04 to 0.19)	0.201	0.05 (−0.01 to 0.10)	0.103	−0.17 (−0.43 to 0.10)	0.229

**Table 6:** Patient satisfaction at T_pre_, T_5_, T_60_, and T_180_, and significant differences between timepoints.

	T_pre_% in Agreement	T_5_% in Agreement	T_60_% in Agreement	T_180_% in Agreement
Feelings				
Presence of shame	36.1	2.6 *	1.0 *	1.3 *
Self-confidence decreased	1.7	2.6 *	1.0 *	1.3 *
Visible being partially edentulous	50.4	0.9 *	1.9 *	1.3 *
Function				
Evade eating with the edentulous zone/implant	62.2	0.9 *	2.9 *	1.3 *
The ability to chew is decreased	62.7	7.0 *	8.7 *	1.3 *
Implant does influence the speech	-	7.0	4.9	1.3
Implant does influence the taste		2.6	1.0	1.3
Esthetics				
Not satisfied with the color of the crown	-	5.4	5.8	0.0
Not satisfied with the contour of the crown	-	7.0	1.9	2.6
Not satisfied with the color of the mucosa around the crown	-	6.5	2.9	1.3
Not satisfied with the contour of the mucosa around the crown	-	11.8	14.6	5.1
Overall satisfaction (0–10, P25 to P75)	5.2 (4.0 to 6.5)	9.0 (8.0 to 10.0) *	9.0 (8.0 to 10.0) *	9.0 (9.0 to 10.0) *

* *p* < 0.001 compared to the pre-treatment scores following the appropriate statistical tests after Bonferroni adjustment; P25 to P75: 25th percentile to 75th percentile.

## Data Availability

The original contributions presented in the study are included in the article, further inquiries can be directed to the corresponding authors. The data presented in this study are available on request from the corresponding author.
